# Towards a Machine Learning Driven Trust Management Heuristic for the Internet of Vehicles

**DOI:** 10.3390/s23042325

**Published:** 2023-02-20

**Authors:** Sarah Ali Siddiqui, Adnan Mahmood, Quan Z. Sheng, Hajime Suzuki, Wei Ni

**Affiliations:** 1 School of Computing, Macquarie University, Sydney, NSW 2109, Australia; 2 Data61, Commonwealth Science and Industrial Research Organisation (CSIRO), Marsfield, NSW 2122, Australia

**Keywords:** vehicular ad hoc networks, Internet of Vehicles, machine learning, trust management, supervised learning, unsupervised learning

## Abstract

The rapid proliferation of the emerging yet promising notion of the Internet-of-Vehicles (IoV) has led to the development of a variety of conventional trust assessment schemes to tackle insider attackers. The primary reliance of these frameworks is on the accumulation of individual trust attributes. While aggregating these influential parameters, weights are often associated with each individual attribute to reflect its impact on the final trust score. It is of paramount importance that such weights be precise to lead to an accurate trust assessment. Moreover, the value of the minimum acceptable trust threshold employed for the identification of dishonest vehicles needs to be carefully defined to avoid delayed or erroneous detection. This paper employs an IoT data set from CRAWDAD by suitably transforming it into an IoV format. This data set encompasses information regarding 18,226 interactions among 76 nodes, both honest and dishonest. First, the influencing parameters (i.e., packet delivery ratio, familiarity, timeliness and interaction frequency) were computed, and two feature matrices were formed. The first matrix (*FM1*) takes into account all the pairwise individual parameters as individual features, whereas the second matrix (*FM2*) considers the average of all pairwise computations performed for each individual parameter as one feature. Subsequently, unsupervised learning is employed to achieve the ground truth prior to applying supervised machine learning algorithms for classification purposes. It is worth noting that Subspace KNN yielded a perfect precision, recall, and the F1-score equal to *1* for individual parametric scores, whereas Subspace Discriminant returned an ideal precision, recall, and the F1-score equal to *1* for mean parametric scores. It is also evident from extensive simulations that *FM2* yielded more accurate classification results compared to *FM1*. Furthermore, decision boundaries among honest and dishonest vehicles have also been computed for respective feature matrices.

## 1. Introduction

The emerging necessity and ever-increasing demand for mobility brought forth a tremendous growth in the number of vehicles, especially in urban areas, globally [1]. In accordance with the reported numbers by the Australian Bureau of Statistics as of 31 January 2021, Australia alone had about 20.1 million registered road vehicles [2]. Globally, this number is expected to rise close to 2.8 billion by 2036 [3]. This escalating count has resulted in numerous challenges, e.g., increasing road fatalities from car crashes and inconvenience from traffic congestion. The leading mortality cause among the younger population (i.e., people from 5 to 29 years old) reported by the World Health Organization is road accidents, and these fatalities accumulate to about 1.3 million annually which makes the daily count nearly 3700 people [4,5]. This demands sophisticated and groundbreaking methodologies for traffic management and control. The technologically advancing and expanding industry of connected vehicles is transforming the conception of transportation through continued enhancements in intelligent transportation systems (ITS) for improved road safety and efficient traffic flows by alleviating road accident risks and traffic congestion [6,7]. In a traffic management system, a massive volume of data acquired by sensors, both internal (i.e., the sensors embedded in modern vehicles) and external (i.e., the sensors installed in the surroundings), is analyzed and processed. These sensors rely upon data sharing among themselves via the Internet of Things (IoT), composed of interconnected computing devices sharing information regarding themselves and their immediate ambiance to build intelligent networks [8].

**Figure 1:**
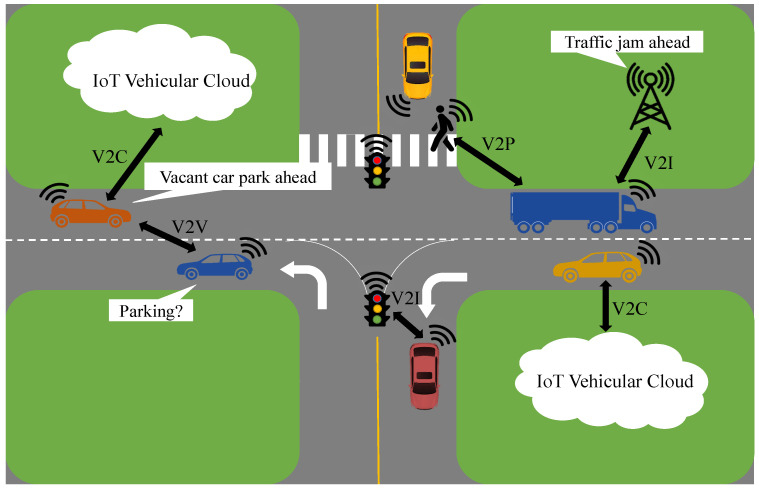
An Overview of Vehicle-to-Everything Communication in an IoV Landscape.

Vehicular Ad hoc Networks (VANETs), evolved from Mobile Ad hoc Networks (MANETS), play a significant role in mitigating problems associated with road traffic in urban areas. The integration of big data, cloud/edge computing and the IoT is motivating the progression of VANETs into the conception of the Internet of Vehicles (IoV) [9,10]. Intelligent vehicles linked together exploit vehicle-to-everything (V2X) communications to ensure road safety and efficient traffic flows, thus assisting cutting-edge transport and road mobility [11]. Through V2X communications, vehicles share data with one another (i.e., vehicle-to-vehicle (V2V)), infrastructure in their vicinity (i.e., vehicle-to-infrastructure (V2I)), personal devices (i.e., vehicle-to-pedestrian (V2P)), embedded sensors (i.e., vehicle-to-sensor (V2S)) and the cloud (i.e., vehicle-to-cloud (V2C)) via wireless media [12,13,14,15] as depicted in Figure 1. These communications are typically applied in (1) safety-critical application scenarios, e.g., road congestion, crash avoidance and collision notification, (2) non-safety (infotainment) application scenarios, e.g., navigation, anti-theft and entertainment [13]. Road safety and traffic management is significantly improved by combining these communications and the abilities of vehicles equipped with onboard sensors to generate obstacle, hazard, collision and congestion warnings and notifications [16]. The critical nature of vehicular applications, particularly safety-critical applications, demands safe and reliable data sharing. Nevertheless, vehicular networks are highly susceptible to both outsider attacks, wherein the adversary is not an authenticated user of the network, and insider attacks, where the attacker is a legitimate participant of the network. These vehicles with their malevolent intentions are capable of introducing unnecessary delays while forwarding, forging and/or altering safety-related information, causing accidents and endangering human lives [17,18]. An extensive study of the existing research exhibits that several solutions for vehicular network security employing cryptography have been presented. Nonetheless, cryptographic solutions on their own only address outsider attacks [19]. Lately, the paradigm of trust, which is the belief or confidence of a vehicle in another vehicle, has been applied to mitigate insider attacks [20]. Trust management schemes rely on trust attributes, i.e., features and characteristics of the network and the communication among vehicles that impact the credibility of these vehicles reflected by their respective trust scores.

While accumulating these trust values, relevant weights reflecting the importance of each influencing parameter are associated with respective parameters to generate intuitive and accurate trust scores. However, translating the said significance into a numeric weight, i.e., weight quantification, is a challenging task. This brings forth the need to study the impacts of contributing parameters individually towards trust computation. Once the final trust has been evaluated, an optimal steady misbehavior detection threshold is selected. The quantification of the specific threshold is of considerable importance as setting the decision boundary extremely low could result in malicious vehicles staying in the network for too long and subsequently damaging the network operations, whereas defining a relatively higher value for the same may lead to trustworthy vehicles being eliminated from the vehicular network.

Machine learning, a prominent flavor of artificial intelligence, is one of the most effective and powerful tools to develop systems in an adaptive and predictive manner and has been widely employed in traditional wireless and vehicular networks. Vehicular networks are highly dynamic in nature, and so, the data-driven techniques aid machine learning to tackle the challenges experienced by traditional solutions for these ever-changing networks [21].

Machine learning has been extensively and effectively applied in various domains, e.g., healthcare, robotics, transportation, computer vision, etc., and it primarily focuses on developing systems with intelligence to be able to work in complex environments. It relies on identifying patterns and inherent structures by analyzing large volumes of data. Being a data-centric methodology, definite suppositions/assumptions have not been imposed on the dissemination of data which provides resilience to process miscellaneous data originating from diverse sources. In vehicular networks, it offers a variety of tools to utilize and help acquire data from heterogeneous sources enabling the system to take well-informed decisions and deliver services, e.g., traffic control and forecasting, in addition to mitigating issues related to communication. Machine learning algorithms are categorized as unsupervised learning algorithms, supervised learning algorithms and reinforcement learning algorithms. The process usually involves two phases, (1) the training phase, wherein training data are utilized for training the specific algorithm in order to generate output, and (2) the testing phase, wherein the testing data are used with the trained model to yield results.

Supervised learning requires a data set with labels or ground truth representing different classes of data [22]. These labels can be either discrete (i.e., for classification) or continuous (i.e., for regression). Subsequently, a part of this data is utilized for training purposes and the other part is for classification or regression testing. The objective of this type of learning is to map out the decision space from the input feature space. In other words, it estimates the mapping function so that it can be applied to any future data in order to generate results. The larger the volume of the data, the more accurate the mapping function gets. On the contrary, unsupervised learning makes use of data without labels or ground truth. It is often not possible to acquire a large volume of labelled data in certain domains, and unsupervised learning helps with describing data samples effectively in such data sets by identifying underlying variables and/or structures by employing Bayesian inference. Clustering is one of the most common variants of unsupervised learning, wherein multiple data samples are grouped together based on the similarities in their features, and each group is termed as a cluster [23].

Accordingly, the primary focus of this paper is to exploit machine learning to tackle the challenge of effective weights assignment and optimal misbehavior detection threshold in vehicular networks. To realize this, a real IoT data set has been transformed into the IoV format and subsequently employed to compute the feature matrix. This feature matrix encompasses four parameters, i.e., *packet delivery ratio*—delineating the throughput between the trustor and the trustee, *familiarity*—depicting how well the trustor knows the trustee, *timeliness*—manifesting the freshness of the interaction among a trustor and a trustee and *interaction frequency*—defining how frequently the trustor and the trustee interacted with one another. Two different approaches have been used to compute the mentioned feature matrix: (a) all four stated parameters computed for a trustee by each trustor are treated as individual features, and (b) the mean of each of these four parameters computed for a trustee by all of the trustors is regarded as a collective feature. The labeling process has been carried out on the generated feature matrix prior to the application of different machine learning algorithms. For the classification of vehicles into honest and dishonest vehicles, support vector machine (SVM), k-nearest neighbors (KNN), ensemble subspace KNN and subspace discriminant were employed. Simulation results demonstrated that more precise results have been yielded via mean parametric scores contrary to the results achieved by taking into consideration the parametric score of each trustor for a single trustee on an individual basis. Please note that the focus of the proposed trust management model is employing supervised learning for categorization of vehicles into trustworthy and malicious vehicles. In order to apply classification algorithms on the computed feature matrices, ground truth is essential. Accordingly, unsupervised learning methodologies have been utilized for labelling purposes only.

The remainder of this paper is organized as follows. Section 2 illustrates the existing state of the art in the subject domain, Section 3 delineates the envisaged system model, Section 4 discusses the simulation results, whereas Section 5 concludes the paper.

## 2. Related Work

A detailed glimpse of the existing literature exhibits several research studies proposing diverse *trust management models* and *intrusion detection frameworks* for malicious vehicle identification and subsequent eradication from the vehicular network. The trust evaluation model presented in [24] relies on a job marketing signaling scheme to encourage cooperative behavior amongst different vehicles in a network. Credit is allocated to each individual vehicle in a vehicular network, and in the event of a dishonest activity by a vehicle, its credit score is reduced by an amount depending on the cost of the attack to penalize the vehicle and discourage malevolent behavior. Similarly, every time a vehicle demonstrates constructive participation, this credit is subsequently increased to promote the node’s participation and its cooperation with other participating vehicles in the network. However, the authors did not discuss the quantification of several coefficient values which affects the preciseness of reward as well as trust evaluations. Moreover, the quantification of weights utilized to reflect historical observation influence has not been addressed. A decentralized scheme utilizing fuzzy logic has been presented by the authors in [25] to tag the dishonest behavior of a vehicle. To evaluate a target vehicle, the suggested framework combines the experience an evaluator has had with that target vehicle and the recommendations received from its neighbors regarding the same vehicle. In addition to the directly connected trustees, the trustors in the proposed model evaluate other vehicles that are not directly connected to the trustor by employing reinforcement learning. The authors, however, did not include any explanation regarding the quantification of the values predefined for the smoothing factor while computing components for direct trust, or learning rate and discount factor while assessing the indirect trust. Moreover, employing fuzzy logic may result in imprecise evaluations.

In [26], the authors envisaged a decentralized privacy-preserving trust assessment framework employing the notion of blockchain. The model dissociates the public key from the vehicle’s true identity during the issuance and revocation of relevant certificates by the certification authority to accomplish anonymity. The blockchain stores all the messages and each vehicle is evaluated based on the information disseminated by it to discourage misbehavior. In order to alleviate misconduct in vehicular networks, an intrusion detection system (IDS) is a noteworthy solution. The stated system employs anomaly and signature-reliant schemes for the identification of mischievous activities and entities. However, the quantification or formulation of the reward as well as the penalty coefficients have not been discussed. In [27], the authors proposed a distributed and cooperative IDS to guarantee private collaboration by employing privacy-preserving distributed machine learning. This framework promotes collaboration wherein all participating vehicles share the training data as well as the corresponding ground truth with one another. This cooperative behavior enhances the quality, cost-efficacy and scalability of the entire system. In addition, the ADMM (i.e., alternating direction method of multipliers) algorithm is utilized to provide a distributed classification solution as well. The authors in [28] suggested an IDS that examines traffic, utilizes a deep belief network for simplification of data dimensionality and segregates legitimate and counterfeited service requests. Moreover, a service-specific grouping has been implemented which guarantees the availability of cloud services at all times. This helps in ensuring both the quality of service and the quality of experience. However, the authors of the envisaged trust management scheme based on supervised learning were able to achieve improved results which are discussed in the later sections of this manuscript. The authors in [29] presented an IDS by combining the support vector machine and the promiscuous mode in a bid to create the trust table for the prevention and detection of attacks. In the proposed model, every vehicle observes its neighbor for dishonest behavior. owever, the authors of the envisaged trust management scheme based on supervised learning were able to achieve improved results which are discussed in the later sections of this manuscript. Similarly, diverse attacks have been introduced in [30] by altering safety messages shared among vehicles, and subsequently, identified various malicious (active) attacks by extracting distinguishing features and employing machine learning techniques. However, the authors of the envisaged trust management scheme based on supervised learning were able to achieve improved results which are discussed in the later sections of this manuscript.

Gao et al. [31] proposed a hybrid trust assessment scheme that combines the direct and the recommendation trust to evaluate the integrated trust of a vehicle relying on historical information regarding interactions among different vehicles. Moreover, Bayesian inference has also been employed while computing the direct trust and penalties have been imposed dynamically. In addition, the notion of a sliding time window has also been introduced while computing the trust values. While computing the integrated trust score, a weighted average of the direct trust and the recommendation trust is calculated. However, the weights utilized remain unexplained which influences the quantification of the final trust value. Furthermore, the application-specific reliability and the related trust trends have not been discussed. Similarly, Zhang et al. [32] also introduced blockchain to assess the reliability of the information exchanged among vehicles, identify dishonest vehicles disseminating malicious information and subsequently impose penalties that negatively impact the reputation of such vehicles. However, the quantification and the decision on the values of the weights defined for computing the credibility of the data, penalty, reward, and the influence on the reputation score, etc., remain unexplained. Furthermore, the authors did not discuss the time-based trends of the credibility of the information dispersed by vehicles.

Ahmad et al. [33] presented a multi-step hybrid trust evaluation framework that evaluates the credibility of the sender prior to the reliability of the information disseminated by that vehicle provided that the particular vehicle is identified as trustworthy. Several parameters have been defined to perform the aforementioned evaluations, e.g., the antenna heights of both the trustor and the trustee, the distance among them, the quality of information, role-dependent trust and the effective distance. However, the quantification of the predefined values for most of these parameters, the penalty and reward factors, along with the time-based variations in the evaluations have not been discussed by the authors. Similarly, Zhang et al. [34] presented an attack-resistant trust evaluation model that computes the vehicles’ local trust by employing Bayesian inference prior to evaluating the global trust of the same vehicles utilizing the TrustRank algorithm. Moreover, parameters related to the behavior of the vehicle’s driver and the vehicle itself are taken into account while computing the above-mentioned trust scores. The quantification of the parameters and those of the associated weights have been discussed in detail. However, the authors employed Bayesian inference which relies heavily on prior probabilities and as opposed to machine learning based pattern recognition, it is able to utilize casual influences only identified by the developer.

Tahani et al. [35] suggested a trust assessment scheme relying on blockchain technology that takes into consideration only the reliability of the events to calculate the pairwise local trust among vehicles. Once local trusts from all monitoring vehicles have been computed, the global trust for each vehicle is computed by aggregating the initial trust and the sum of all local trusts assigned to a particle vehicle prior to averaging it out. The authors did not discuss the quantification of the increment value associated with the local trust and while taking the average for global trust, all individual local trusts evaluated by all monitors have been assigned equal weights. Similarly, Muhsen et al. [36] proposed a fuzzy logic driven trust evaluation framework that takes into account the neighbor recommendations, direct trust and the message accuracy as parameters to compute trust scores. For recommendations, opinions of both neighboring vehicles and the neighboring roadside units (RSUs) are considered, whereas for direct trust, historical interactions among the particular RSU and the vehicle being evaluated are taken. The message reliability is evaluated by the RSU on the basis of the events reported by trustworthy vehicles. However, the work lacks discussion on how the trust evaluations by neighboring vehicles and the direct trust by RSUs are quantified. Moreover, the quantification of threshold for the number of interactions for referees and the weight for individual transactions is not addressed. Furthermore, the RSU is responsible for performing all trust evaluations and verification of events, this builds a centralized architecture which is not practical as if the RSU fails, the individual vehicles will not be able to evaluate the reliability of peer vehicles.

The existing literature has already demonstrated some significant contributions by applying numerous machine learning techniques. Nevertheless, they only rely on the conventional factors in the trust assessment process and the impact of the influential parameters *(i.e., packet delivery ratio, familiarity, timeliness and interaction frequency)* on the trust assessment and aggregation process has been completely ignored. Furthermore, the introduction and quantification of weights associated with influential individual parameters have not been discussed along with a lack of discussion on the misbehavior detection threshold formalization and quantification. Accordingly, the authors employed machine learning techniques to aggregate contributing trust parameters and to identify misbehavior without the need to manually assign weights or predefine the detection threshold.

## 3. System Model

We hereby propose a trust evaluation framework reliant on machine learning techniques for the identification of dishonest vehicles for elimination from the vehicular network in order to prevent them from causing any further harm and to conserve precious network resources. The developed system model encompasses two key steps. First, unsupervised learning algorithms have been utilized to cluster and label the data prior to employing supervised learning algorithms for the classification of vehicles into two categories, i.e., *untrustworthy* and *trustworthy* as mentioned in Table 1.

The simulations are performed for a vehicular network (or a cluster) comprising *N* vehicles. Every vehicle *i*, wherein i=1,…,N has one-hop neighbors *j*, wherein j=1,…,N and (i≠j), and evaluates them, i.e., *i* is the trustor, and *j* is the trustee. The evaluation transpires on the basis of four parameters—packet delivery ratio ($PDR_{i,j}$), familiarity ($FMR_{i,j}$), timeliness ($TML_{i,j}$) and interaction frequency ($IFR_{i,j}$). The parameter values vary in the range of zero and one, wherein, zero represents the lowest correlation between a pair of a trustor and a trustee, whereas one signifies the highest correlation of the said pair.

### 3.1. Data Set and Feature Extraction

For the proposed system model, we have used an IoT data set from CRAWDAD (https://ieee-dataport.org/open-access/crawdad-thlabsigcomm2009, accessed on 20 February 2023) by suitably transforming it into an IoV format. The cited data set has been widely adopted for trustworthiness in social IoT as it includes trust factors pertinent to humans as well as devices/nodes. Moreover, it was devised for evaluating what engages humans or entities (i.e., nodes in the IoT or vehicles in an IoV) and what community they are responsive to. The proposed trust management model has been evaluated using MATLAB simulations for all 76 vehicles. We defined four scoring parameters, i.e., *packet delivery ratio*, *familiarity*, *timeliness* and *interaction frequency* for evaluating each node in the network.

*Packet Delivery Ratio (PDR)*—The packet delivery ratio (0≤PDR≤1) is the degree of how well a trustor is connected to the trustee. In other words, the packet delivery ratio represents the proportion of the messages received by a trustor and is computed via Equation (1) as:

(1) $$PDR_{i,j}=\frac{M_{i,j}}{M_{i_{j}}}$$

where $M_{i,j}$ is the number of messages sent by the trustee *j* that were successfully received by the trustor *i*, and $M_{i_{j}}$ is the total number of messages sent to *i* by *j*.

*Familiarity (FMR)*—Familiarity (0≤FMR≤1) refers to the degree of how well a trustor knows the trustee. The familiarity is computed via Equation (2) as:

(2) $$FMR_{i,j}=\frac{F_{i,j}}{F_{i}}$$

where $F_{i,j}$ is the number of common friends between both the trustor and the trustee, and $F_{i}$ is the total number of the trustor’s friends.

*Timeliness (TML)*—Timeliness (0≤TML≤1) refers to the degree of how fresh the interaction between a trustor and a trustee is. The timeliness is computed via Equation (3) as:

(3) $$TML_{i,j}=\frac{T_{i,j}}{T_{current}}$$

where $T_{i,j}$ is the time when the interaction between the trustor and the trustee took place, and $T_{current}$ is the current time instance.

*Interaction Frequency (IFR)*—Interaction Frequency (0≤IFR≤1) refers to the degree of how often a trustor interacts with the trustee. The interaction frequency is computed via Equation (4) as:

(4) $$IFR_{i,j}=\frac{\sum I_{i,j}}{\sum I_{i}}$$

where $I_{i,j}$ is the interaction between a trustor and a trustee, and $I_{i}$ is the total number of the trustor’s interactions.

A pairwise, i.e., among a pair of a trustor and a trustee, computation of all four stated parameters is performed and the subsequent scores are recorded in the feature matrix. As mentioned above, two different feature matrices are generated and in the first, each row represents an individual trustee and each column represents an individual parameter (i.e., PDR, FMR, TML and IFR) ascertained pairwise, i.e., among a pair of a trustor and a trustee on an individual basis. In other words, there are *N* = 76 number of rows and 4N−4 number of columns. The described feature matrix (see Equation (5)) is generated with the objective of the impact analysis of each trustor for a trustee against each parameter in the final classification.

(5) $$\begin{array}{c} FM1=\left[\begin{array}{cccc} PDR_{11}\cdots PDR_{1N-1} & FMR_{11}\cdots FMR_{1N-1} & TML_{11}\cdots TML_{1N-1} & IFR_{11}\cdots IFR_{1N-1}\\ \vdots \;\;\ddots \;\;\vdots & \vdots \;\;\ddots \;\;\vdots & \vdots \;\;\ddots \;\;\vdots & \vdots \;\;\ddots \;\;\vdots \\ PDR_{N1}\cdots PDR_{NN-1} & FMR_{N1}\cdots FMR_{NN-1} & TML_{N1}\cdots TML_{NN-1} & IFR_{N1}\cdots IFR_{NN-1} \end{array}\right] \end{array}$$

In the second feature matrix, the rows represent the trustees, i.e., there are *N* = 76 number of rows, and the columns represent the mean of each parameter (PDR, FMR, TML and IFR) computed for each trustee by all the trustors, i.e., there are 4 columns in total. The means for PDR, FMR, TML and IFR are obtained via Equations (6)–(9), respectively. This feature matrix (see Equation (10)) is formed with the intent to classify the vehicles on the basis of their mean parametric scores.

(6) $$PDR_{avg_{j}}=\frac{\sum_{i=1}^{N}PDR_{i,j}}{N-1},\;i\neq j$$

(7) $$FMR_{avg_{j}}=\frac{\sum_{i=1}^{N}FMR_{i,j}}{N-1},\;i\neq j$$

(8) $$TML_{avg_{j}}=\frac{\sum_{i=1}^{N}TML_{i,j}}{N-1},\;i\neq j$$

(9) $$IFR_{avg_{j}}=\frac{\sum_{i=1}^{N}IFR_{i,j}}{N-1},\;i\neq j$$

where $PDR_{i,j}$, $FMR_{i,j}$, $TML_{i,j}$ and $IFR_{i,j}$ is the computed packet delivery ratio, familiarity, timeliness and interaction frequency, respectively, among a pair of a trustor *i* and a trustee *j*.

(10) $$FM2=\left[\begin{array}{cccc} PDR_{avg_{1}} & FMR_{avg_{1}} & TML_{avg_{1}} & IFR_{avg_{1}}\\ \vdots & \vdots & \vdots & \vdots \\ PDR_{avg_{N}} & FMR_{avg_{N}} & TML_{avg_{N}} & IFR_{avg_{N}} \end{array}\right]$$

### 3.2. Clustering and Labeling

Subsequent to the score computation for each parameter (i.e., via Equations (1)–(4), these scores are utilized for the classification of vehicles into two clusters, i.e., *trustworthy* and *untrustworthy*. In order to ascertain these clusters, unsupervised learning algorithms, i.e., *k-means*, *fuzzy c-means*, *hierarchical clustering* and *Gaussian mixture* based envisaged algorithms have been employed and the feature matrices generated previously have been labelled. The primary rationale behind employing diverse unsupervised learning algorithms is to guarantee a credible, reliable and persistent ground truth.

K-means clustering is a partitional method, i.e., it assumes that expressing a given data set in the form of finite groups, known as clusters, having individual selection criteria is achievable. Each group has a group profile, known as the cluster prototype that defines the data points inside that particular group. Partitioning methods rely on the difference or distance between a data point and a group’s prototype. K-means algorithms are recognized as the most well-known and earliest partitional method [37]. Initialization parameters influence these algorithms in addition to assigning in prior to these clusters [38,39,40]. K-means clustering converges fast due to its simplicity [41].

Fuzzy c-means clustering relies on the Euclidean distance between the data points and the centroids. Similar to k-means, fuzzy c-means clustering is also impacted by the initialization parameters. However, they achieve convergence more rapidly as compared to k-means clustering. Due to the symmetric nature of Euclidean distance, its reliance on the Euclidean distance implies that the significance of variables in a given data set is considered to be the same. In this particular clustering algorithm, each data point may belong to multiple groups. Therefore, it is known as a soft clustering method, wherein a probability or likelihood is associated with each data point to be a part of a certain group or cluster [42,43].

Hierarchical clustering employs a bottom-up (i.e., agglomerative) approach or a top-down (i.e., divisive) technique to group data points into hierarchical groups or clusters. It is widely applied to analyze data statistically and for data mining. In this clustering method, it is not a requirement to initiate the number of resulting clusters. However, its computational efficiency is quite high and such methods are not suitable for high-dimensional data sets. Hierarchical clustering differs from the aforementioned partitional methods as they generate a hierarchical breakdown of data instead of separating it into multiple groups depending on the prototype [44].

The Gaussian mixture model is a soft clustering method, i.e., it assigns probabilities or likelihood to each data point to be classified in a specific cluster. It takes preference over k-means as it takes into consideration the variation in the data which refers to the shape of the curve, therefore, is appropriate for elliptical-shaped clusters as well as circular ones. Gaussian mixture models employed for clustering suppose that every data point forms a Gaussian distribution. This clustering technique is believed to outperform other clustering algorithms as it factors in the number of clusters and the position of the same along with the shape [45,46,47].

The cluster close to the origin is tagged as *dishonest* or *malicious*, whereas the one away from the origin is categorized as *honest* or *trusted*. This implies that the vehicles having a higher parametric score are more credible as compared to the vehicles with a lower parametric score. Subsequently, the labels obtained are integrated into the corresponding feature matrix. It is worth noting that the labels in each feature matrix will have different values than the other due to a difference in data points inside these matrices. The algorithm for clustering and labeling via only fuzzy c-means has been specified here for reference purposes (see Algorithm 1). In Algorithm 1, the variable $C_{j}$ represents the centroids of the clusters. The value of *j* depends on the total number of clusters, and it has been defined/fixed as two since the number of desired clusters in the envisaged model is two, i.e., one for trustworthy and the other for malicious (untrustworthy) vehicles. Please note that no algorithm or specific technique has been employed to fix *j* as two. In addition, note that only the algorithm for fuzzy c-means has been presented due to space constraints.
**Algorithm 1** Labeling using Fuzzy C-means     **Input**: FM1/FM2     **Output**: Labels 1:Initialize cluster centers $c_{1}$, $c_{2}$, ..., $c_{j}$ 2:**for**j=1to2**do** 3:    Repeat until convergence: { 4:    Calculate the membership values $w_{ij}$ 5:    $w_{ij}\leftarrow \frac{1}{\frac{\left|\right|X^{\left(i\right)}-c_{j}\left|\right|}{\left|\right|X^{\left(i\right)}-c_{k}\left|\right|}}$ 6:    **for** i=1ton **do** 7:        $a^{\left(i\right)}\leftarrow arg\;min_{c}\;w_{ij}^{m}\;\left|\right|{X^{\left(i\right)}-c_{j}\left|\right|}^{2}$ 8:    **end for** 9:}10:    $K^{\left(j\right)}\left(a,c\right)\leftarrow arg\;min_{j}\;K\left(a,c\right)$11:**end for**12:**for**i=1ton**do**13:    **if** $a^{\left(i\right)}$ close to *origin* **then**14:        $Labels^{\left(i\right)}\leftarrow 1$15:    **else**16:        $Labels^{\left(i\right)}\leftarrow 2$17:    **end if**18:**end for**

### 3.3. Classification Model

Subsequent to the clustering and labeling of data, supervised learning classifiers have been applied to the generated feature matrices for training purposes with a *five-fold cross-validation*. This is for the identification of malicious vehicles by obtaining an optimal decision boundary due to the distinct characteristics. A diverse variety of machine learning algorithms based on *k-nearest neighbors*, *support vector machine* and *ensemble classification models* have been employed.

The k-nearest neighbors (KNN) algorithm is considered one of the simplest techniques and is employed for both regression and classification. It employs the notion of neighborhood proximity, i.e., similarity or distance-based measure is utilized for classification. This means that every new data point is categorized as a part of the same class as that of the nearest neighbor, i.e., the closest data item. The primary goal of this algorithm is to generate a prediction model relying on the training data points and to predict relevant labels for the testing data points. Despite the ease of use, KNN is not suitable for certain applications as it caches the entire set of data points in the memory and is computationally complex and costly [48,49].

Support vector machine (SVM) is a well-known technique and can also be used for both classification and regression. It yields highly accurate results. However, it is more suitable for small data sets due to longer processing times. It works by determining an optimum boundary for data separation relying on the labels, i.e., the ground truth, assigned to these data points. SVM is less susceptible to overfitting, wherein the model fits exactly or too close to a part of the data set, i.e., a small collection of data points. It is capable of supporting high-dimensional data and can be used for linear as well as non-linear classification of data. Among multiple decision boundaries, SVM formulates the one with a maximum distance from the data points in the training data set belonging to any of the classes. The greater the distance, the higher the accuracy and the lower the classification error are [48,50].

Ensemble classification models are generally formulated by combining multiple base classifiers, wherein each classifier individually determines the decision boundaries between different classes by learning patterns from the training data. The classification outcome of these classifiers on the testing dagta are generated by the amalgamation of separate decisions of each of the base classifiers. The results yielded by these ensemble classifiers are more accurate in comparison to the individual base classifiers given that the individual errors of the contributing base classifiers are uncorrelated. The modern techniques to devise ensembles utilize distinct portions of the entire training set to train individual base classifiers resulting in uncorrelated errors. The classification outcome individual base classifiers are consolidated to produce the final decision by employing majority voting or algebraic combiners depending on the form of decisions, i.e., discrete or continuous, respectively [51,52,53].

The algorithm for classification and decision boundary has also been specified (see Algorithm 2).
**Algorithm 2** Classification and Decision Boundary     **Input**: FM1/FM2, Labels     **Output**: Accuracy, Decision boundary1:Initiate *n* classification models with *k* fold validation (*k* = 5)2:**for**z=1ton**do**3:    Find the classification accuracy and decision boundary of each machine learning model4:    $\left[Acc_{z},B_{z}\right]=model_{z}\left(FM1/FM2,Labels,k\right)$5:**end for**

## 4. Simulation Results

This section focuses on the simulation results yielded by applying the envisaged trust model on the data set employing MATLAB.

### 4.1. Clustering and Labeling

**Figure 2:**
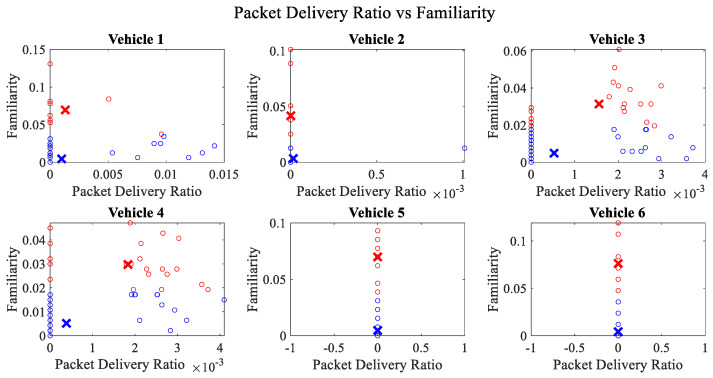
Clustering for labels using unsupervised learning for packet delivery ratio vs. familiarity for vehicles 1−6. The cluster in blue represents untrustworthy vehicles, whereas the cluster in red depicts trustworthy vehicles.

**Figure 3:**
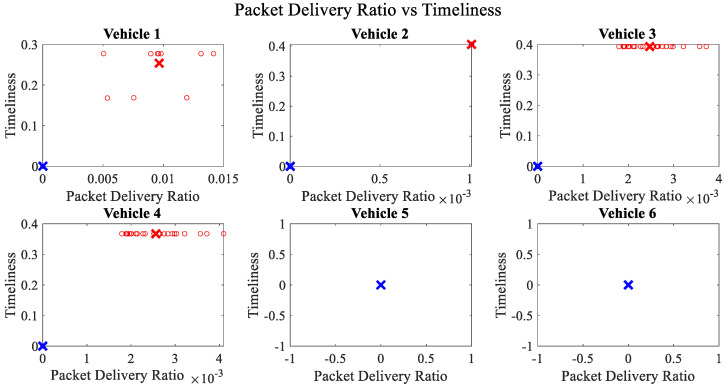
Clustering for labels using unsupervised learning for packet delivery ratio vs. timeliness for vehicles 1−6. The cluster in blue represents untrustworthy vehicles, whereas the cluster in red depicts trustworthy vehicles. The rather empty results for vehicle 5 and vehicle 6 demonstrate that both vehicles have zero values for packet delivery ratio and timeliness with all other vehicles.

**Figure 4:**
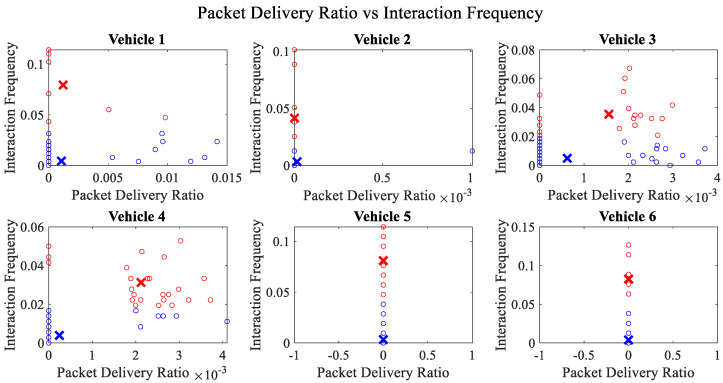
Clustering for labels using unsupervised learning for packet delivery ratio vs. interaction frequency for vehicles 1−6. The cluster in blue represents untrustworthy vehicles, whereas the cluster in red depicts trustworthy vehicles.

**Figure 5:**
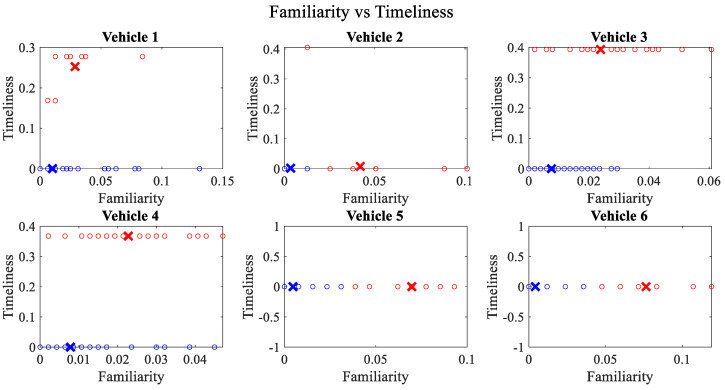
Clustering for labels using unsupervised learning for familiarity vs. timeliness for vehicles 1−6. The cluster in blue represents untrustworthy vehicles, whereas the cluster in red depicts trustworthy vehicles.

**Figure 6:**
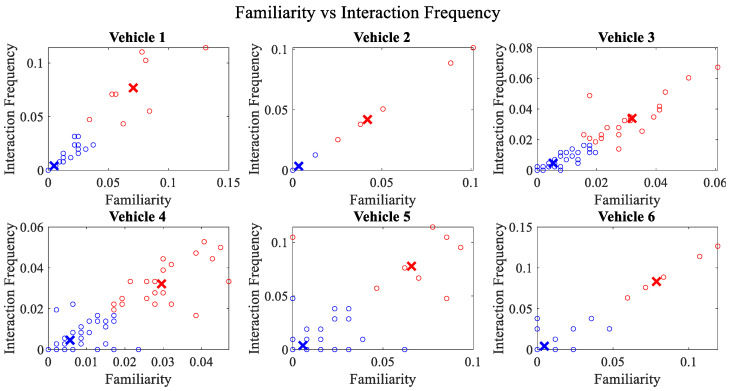
Clustering for labels using unsupervised learning for familiarity vs. interaction frequency for vehicles 1−6. The cluster in blue represents untrustworthy vehicles, whereas the cluster in red depicts trustworthy vehicles.

**Figure 7:**
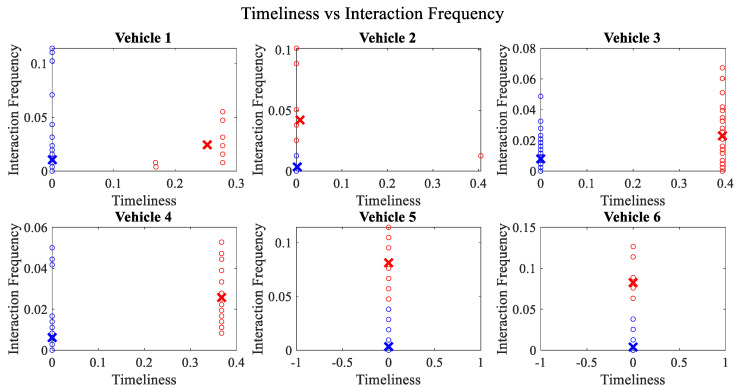
Clustering for labels using unsupervised learning for timeliness vs. interaction frequency for vehicles 1−6. The cluster in blue represents untrustworthy vehicles, whereas the cluster in red depicts trustworthy vehicles.

**Figure 8:**
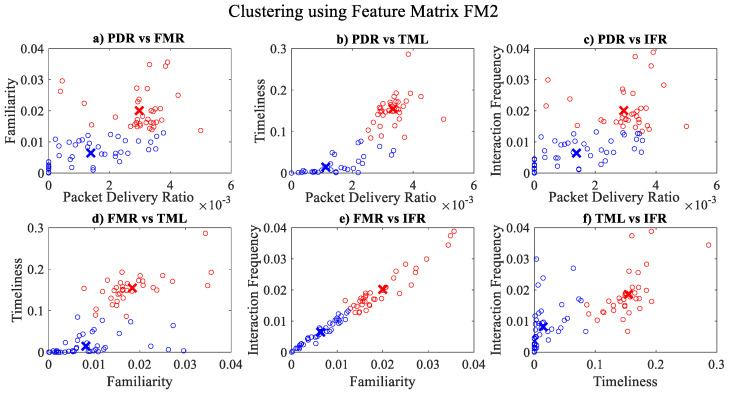
Clustering for labels using unsupervised learning on FM2: (**a**) packet delivery ratio vs. familiarity; (**b**) packet delivery ratio vs. timeliness; (**c**) packet delivery ratio vs. interaction frequency; (**d**) familiarity vs. timeliness; (**e**) familiarity vs. interaction frequency; (**f**) timeliness vs. interaction frequency; the cluster in blue represents untrustworthy vehicles, whereas the cluster in red depicts trustworthy vehicles.

It is pertinent to mention that only the clustering of data points employing fuzzy c-means is illustrated in the figures. The clustering of data points (i.e., vehicles) from *FM1* is presented in Figure 2, Figure 3, Figure 4, Figure 5, Figure 6 and Figure 7 only for vehicles 1–6, whereas the clustering of *FM2* into two clusters is depicted in Figure 8. To facilitate visuality, the clustering for each pair of features is depicted. The vehicles belonging to the cluster closer to the origin are labelled as malicious, whereas the members of the cluster farther from the origin are labelled as trustworthy. In Figure 3, vehicles 5 and 6 exhibit no values as both the PDR and TML of these vehicles with all other vehicles are zero. Therefore, no clustering can be performed.

Figure 4 depicts that both vehicle 5 and vehicle 6 have interacted with other vehicles in the network; however, these interactions have not been successful which is reflected by the packet delivery ratio values in the respective sub-figures. This implies that the labelling decision in these specific sub-figures is relying entirely on the interaction frequency. In Figure 5, it can be seen that vehicle 3 and vehicle 4 have interactions with other vehicles only in two time windows with regards to familiarity. Vehicle 2 in Figure 6 delineates an interesting behavior with equal values of familiarity to the corresponding interaction frequencies. Similar to Figure 4, Figure 7 shows that vehicle 5 and vehicle 6 have zero timeliness with all other vehicles due to zero packet delivery ratio which shows that in these sub-figures, the labelling decisions are based on interaction frequency only.

**Figure 9:**
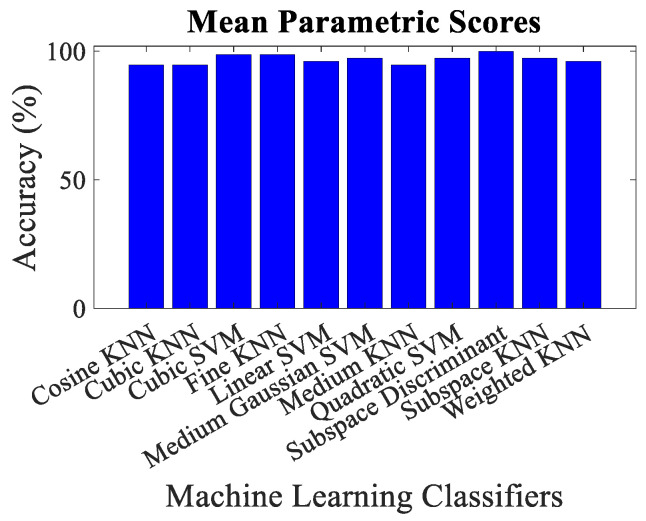
Classification accuracy for mean parametric scores using different machine learning classifiers.

**Figure 10:**
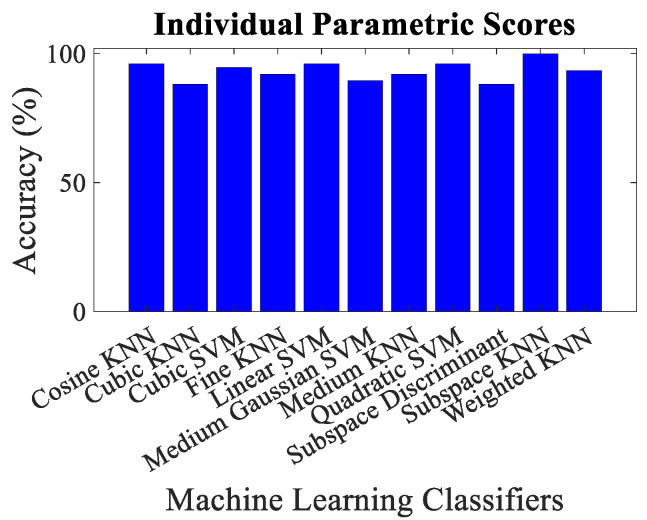
Classification accuracy for individual parametric scores using different machine learning classifiers.

The overall accuracy, malicious node classification accuracy, precision, recall, F1-score and decision boundary using each feature matrix for each classifier have been computed for performance evaluation purposes. Simulation results revealed that the classification via mean parametric scores yielded more accurate results, as shown in Figure 9 in contrast to the one which takes into account the parametric score of each trustor for a trustee on an individual basis, as shown in Figure 10. It could be observed that the minimum overall classification accuracy while taking mean parametric score is yielded by Cosine KNN, Cubic KNN and Medium KNN as 94.7%, whereas while using individual parametric scores, the minimum overall classification accuracy is yielded by Cubic KNN and Subspace Discriminant and is found to be 88.2%. It is noteworthy that only the lowest accuracies have been mentioned here to emphasize the effectiveness of the proposed trust management scheme. It is also pertinent to highlight that the best malicious vehicle classification result of the proposed trust management model is yielded by taking the mean parametric scores and via Subspace Discriminant.

### 4.2. Classification Model

**Figure 11:**
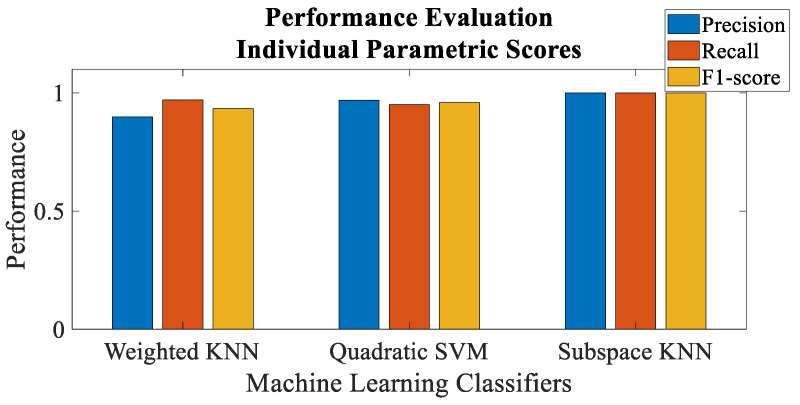
Performance evaluation of individual parametric scores for malicious vehicle classification.

**Figure 12:**
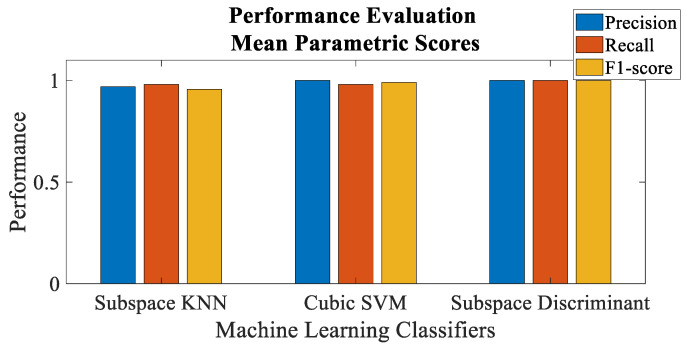
Performance evaluation of mean parametric scores for malicious vehicle classification.

**Figure 13:**
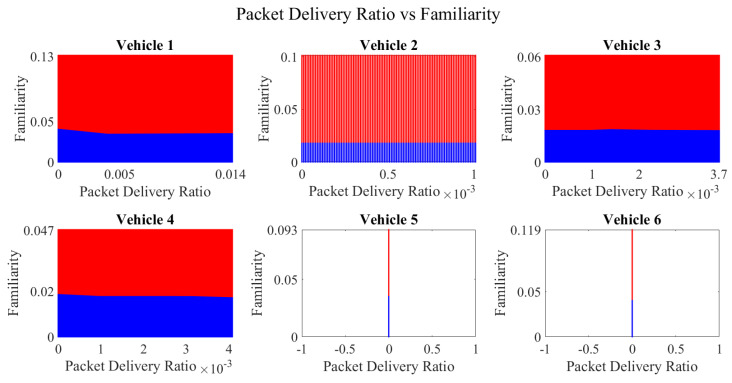
Decision boundary for packet delivery ratio vs. familiarity for vehicles 1−6. Boundary for *untrustworthy vehicles* is depicted in blue, whereas red manifests the *trustworthy* vehicles’ region.

**Figure 14:**
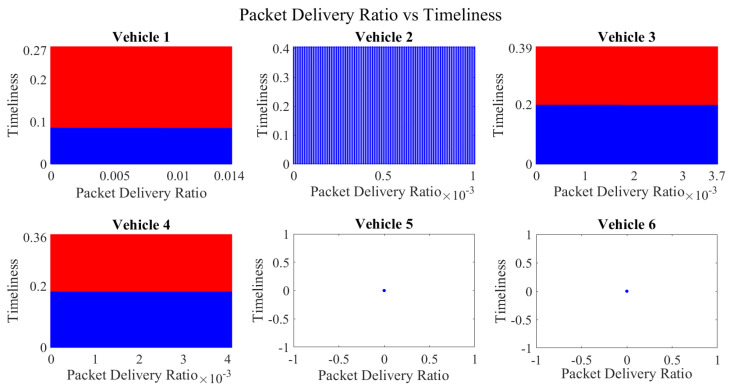
Decision boundary for packet delivery ratio vs. timeliness for vehicles 1−6. Boundary for *untrustworthy vehicles* is depicted in blue, whereas red manifests the *trustworthy* vehicles’ region. The empty results for vehicle 5 and vehicle 6 demonstrate that both vehicles have zero values for packet delivery ratio and timeliness with all other vehicles.

**Figure 15:**
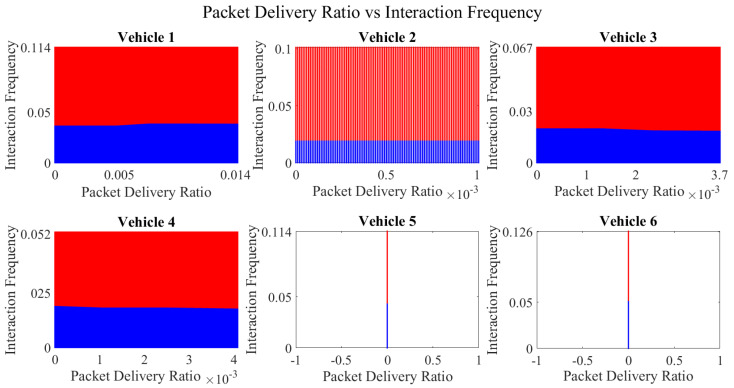
Decision boundary for packet delivery ratio vs. interaction frequency for vehicles 1−6. Boundary for *untrustworthy vehicles* is depicted in blue, whereas red manifests the *trustworthy* vehicles’ region.

**Figure 16:**
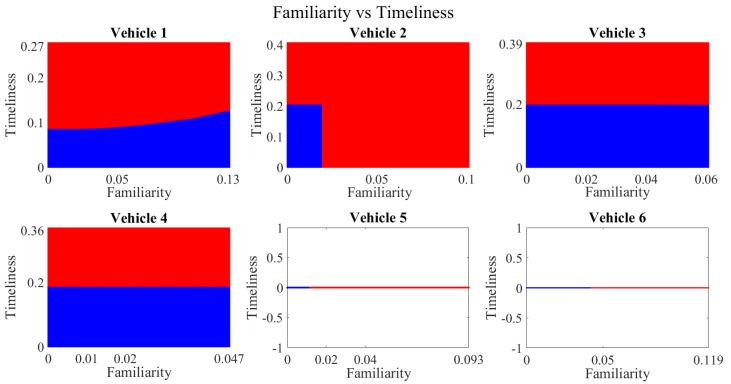
Decision boundary for familiarity vs. timeliness for vehicles 1−6. Boundary for *untrustworthy vehicles* is depicted in blue, whereas red manifests the *trustworthy* vehicles’ region.

**Figure 17:**
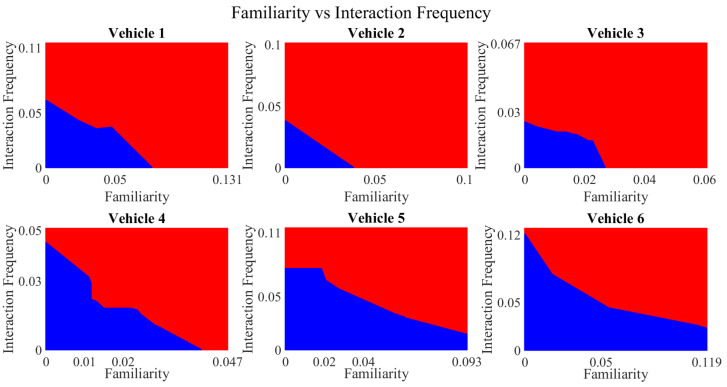
Decision boundary for familiarity vs. interaction frequency for vehicles 1−6. Boundary for *untrustworthy vehicles* is depicted in blue, whereas red manifests the *trustworthy* vehicles’ region.

**Figure 18:**
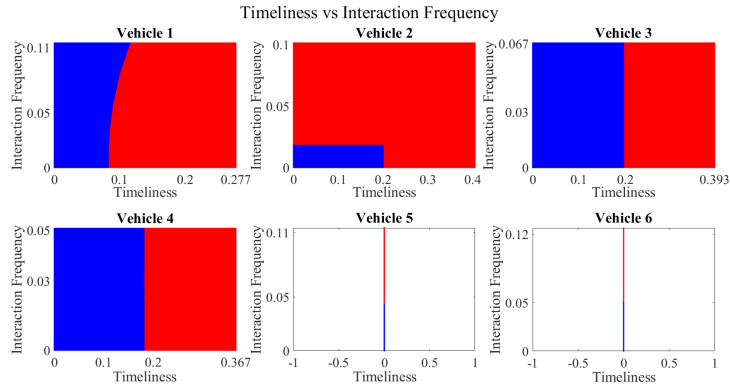
Decision boundary for timeliness vs. interaction frequency for vehicles 1−6. Boundary for *untrustworthy vehicles* is depicted in blue, whereas red manifests the *trustworthy* vehicles’ region.

**Figure 19:**
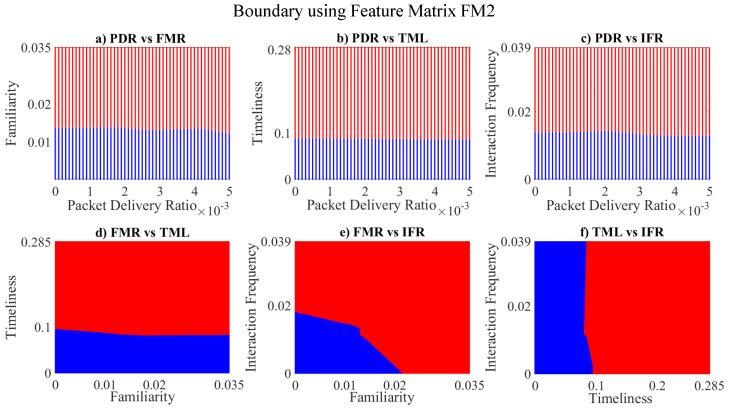
Decision boundary for: (**a**) packet delivery ratio vs. familiarity; (**b**) packet delivery ratio vs. timeliness; (**c**) packet delivery ratio vs. interaction frequency; (**d**) familiarity vs. timeliness; (**e**) familiarity vs. interaction frequency; (**f**) timeliness vs. interaction frequency; boundary for *untrustworthy vehicles* is depicted in blue, whereas red manifests the *trustworthy* vehicles’ region.

Figure 11 and Figure 12 depict the performance evaluation of the envisaged trust model with respect to malicious node classification in terms of precision, recall and F1-score for individual and mean parametric scores, respectively. Precision is actually defined as the accuracy of the model to classify malicious nodes as malicious, whereas recall is the proportion of the malicious nodes that have been correctly identified. F1-score represents the weighted mean of the two. All the three performance evaluation metrics mentioned above range from 0 to 1, i.e., 0 represents the *worst* and 1 represents the *best* performing model. It can be noted that Subspace KNN yields a perfect precision, recall and the F1-score equal to one for individual parametric scores, whereas Subspace Discriminant returns an ideal precision, recall and the F1-score equal to one for mean parametric scores. Figure 13, Figure 14, Figure 15, Figure 16, Figure 17 and Figure 18 demonstrate the pair-wise decision boundaries between the *trustworthy* and *untrustworthy* vehicles using Subspace KNN classifier for individual parametric scores, whereas Figure 19 illustrates the pair-wise decision boundaries between the *trustworthy* and *untrustworthy* vehicles using Subspace KNN classifier for mean parametric scores. In Figure 13 and Figure 15, it can be seen that the honest and malicious regions for vehicle 2 are broken, and this is because the pairwise data points for familiarity and packet delivery ratio are far apart. In Figure 14, the rather empty results demonstrate that both these vehicles have zero values for packet delivery ratio as well as timeliness for other vehicles. It can also be seen that vehicle 2 seems to be trustworthy for most of the vehicles for almost all of the parametric pairs as the red regions are significantly bigger than the blue.

Table 2 summarizes the comparison of the envisaged trust management heuristic to the recent state of the art in vehicular trust management. It can be seen that the proposed methodology outperforms the remaining techniques.

## 5. Conclusions

In this paper, we have proposed a distributed trust management model that takes into account the notion of *packet delivery ratio*, *familiarity*, *timeliness* and *interaction frequency* amongst the vehicles and employs supervised learning to aggregate/accumulate a vehicle’s trust score to identify and subsequently eradicate multiple malicious vehicles in real-time by computing an optimal trust threshold. Simulation results manifest the significance of the selected feature parameters in the classification of dishonest vehicles. Prior to applying supervised learning, two different feature matrices were generated, one by averaging each parameter for all vehicles and the other with each parameter calculated for each pair of vehicles individually. The classification accuracy for each of the matrices has been evaluated, and the decision boundaries are estimated. In the future, the authors intend to further explore trust influencing parameters along with dynamic attack models. Moreover, they intend to work on a purpose-built IoV simulator to generate large trust-based IoV data sets in the future.

## Figures and Tables

**Table 1:** Classification Groups.

Groups	Labels
1	Untrustworthy
2	Trustworthy

**Table 2:** Result comparison with state-of-the-art trust management frameworks.

Ref.	Data Set Size	Accuracy (%)	Recall (%)	Precision (%)	F1-Score (%)
[28]	40 vehicles	99.5	—	—	—
[29]	50 vehicles	—	96.75	100	98.35
[30]	4528 samples	—	92.9	98.9	95.81
[54]	273,097 records	96.93	90.4	98.8	94.41
[55]	80 vehicles	—	89.3	91.8	90.53
[56]	100 vehicles	—	93.5	94	93.75
Proposed	76 vehicles	100	100	100	100
